# The impact of estimated tumour purity on gene expression-based drug repositioning of Clear Cell Renal Cell Carcinoma samples

**DOI:** 10.1038/s41598-019-39891-y

**Published:** 2019-02-21

**Authors:** Karel K. M. Koudijs, Anton G. T. Terwisscha van Scheltinga, Stefan Böhringer, Kirsten J. M. Schimmel, Henk-Jan Guchelaar

**Affiliations:** 1 Department of Clinical Pharmacy & Toxicology, Leiden University Medical Centre, Leiden, The Netherlands; 2 Department of Medical Statistics, Leiden University Medical Centre, Leiden, The Netherlands

## Abstract

To find new potentially therapeutic drugs against clear cell Renal Cell Carcinoma (ccRCC), within drugs currently prescribed for other diseases (drug repositioning), we previously searched for drugs which are expected to bring the gene expression of 500 + ccRCC samples from The Cancer Genome Atlas closer to that of healthy kidney tissue samples. An inherent limitation of this bulk RNA-seq data is that tumour samples consist of a varying mixture of cancerous and non-cancerous cells, which influences differential gene expression analyses. Here, we investigate whether the drug repositioning candidates are expected to target the genes dysregulated in ccRCC cells by studying the association with tumour purity. When all ccRCC samples are analysed together, the drug repositioning potential of identified drugs start decreasing above 80% estimated tumour purity. Because ccRCC is a highly vascular tumour, attributed to frequent loss of VHL function and subsequent activation of Hypoxia-Inducible Factor (HIF), we stratified the samples by observed activation of the HIF-pathway. After stratification, the association between estimated tumour purity and drug repositioning potential disappears for HIF-activated samples. This result suggests that the identified drug repositioning candidates specifically target the genes expressed by HIF-activated ccRCC tumour cells, instead of genes expressed by other cell types part of the tumour micro-environment.

## Introduction

Tumours of metastatic clear cell renal carcinoma (ccRCC) patients typically become resistant to available treatments within 1.5 years^1^. To discover new potentially therapeutic drugs against ccRCC within drugs already prescribed for diseases (drug repositioning), we previously developed an individualised drug repositioning approach based on the gene expression profiles of over 500 ccRCC tumours generated using bulk RNA-Seq by The Cancer Genome Atlas (TCGA)^2^.

With bulk RNA-seq the gene expression of all cell types present in the sample is measured simultaneously^3^. The presence of non-cancerous cells might be an especially big concern for ccRCC samples, as ccRCC estimated tumour purity was ranked the third most impure tumour type out of the total 21 solid tumours analysed, despite the lower mutational burden typical for other highly impure cancers^4^. The relatively low content of cancerous cells in ccRCC tumours is likely due to the high degree of vascularity often observed in ccRCC tumours^5,6^. This hypervascularity is attributed to the frequent inactivation of the Von Hippel-Lindau gene, which leads to activation of the Hypoxia Inducible Factor (HIF) pathway and the subsequent release of vascularizing growth factors: Vascular Endothelial Growth Factor (VEGF), platelet-derived growth factor beta (PDGFβ), and transforming growth factor alpha (TGFα)^7^.

Aran *et al*. reported in their systematic pan-cancer analysis of TCGA tumour sample purity that variation in estimated tumour purity can significantly influence the results of differential gene expression analyses^4^. After adjusting for estimated tumour purity, on average 14% of differentially expressed genes lost statistical significance and 11% of expressed genes were now shown to be statistically differentially expressed when they were not before adjustment^4^. Depending on whether tumour purity is seen as a factor that needs to be corrected, such as in the case when only tumour cells are of interest, the differential gene expression profile can therefore change drastically.

Computational drug repositioning methods which rely on transcriptomic data commonly use this data type without accounting for the potential influence of tumour sample composition. The drug repositioning method we used, gene expression signature reversal, functions by searching for drugs which can normalize the genes which are differentially expressed in the tumour tissue (i.e. up- or downregulated compared to the surrounding normal tissue). Specifically, drugs which can get tumour gene expression closer to that to normal tissue are considered potentially therapeutic drugs for this tumour. However, if genes are incorrectly classified as differentially expressed due to the confounding presence of non-cancerous cells present in the sample, it naturally follows that this can reduce the predictive validity of the procedure if the intention is to target the tumour cells with the drug. Excluding these drugs early on would therefore save vital time and money spent on laboratory validation experiments to determine whether the drug is likely to be safe and effective at clinically tolerated dosing regimens. Furthermore, while we considered all differentially expressed genes to be of interest in our initial drug repositioning analysis, the same issue comes up when only genes of a specific biological pathway are of interest if these genes are sufficiently expressed in the surrounding non-cancerous cells.

The goal of the current study is to investigate whether the previously identified drug repositioning candidates are indeed expected to target the dysregulated genes expressed by ccRCC tumour cells and are not systematically influenced by variation in tumour purity between the ccRCC samples.

## Results

### Connectivity scores versus estimated tumour purity

Tumour purity estimations and connectivity scores (requiring ≥10 differentially expressed genes) were available for 529 tumour sample gene expression profiles, of which 521 also passed the independent Toil quality control^8^.

**Figure 1:**
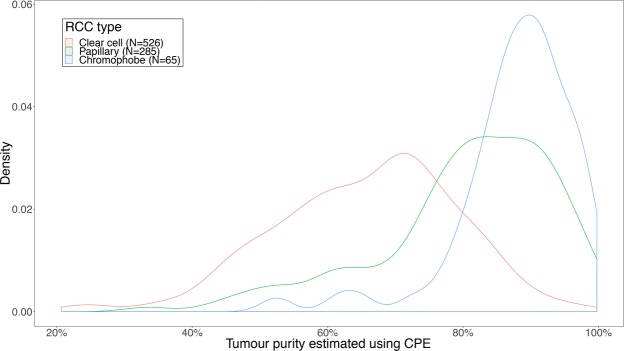
Distribution of estimated tumour purity of clear cell, papillary and chromophobe RCC samples for which estimated tumour purity was available and for samples which passed Toil quality control. 1/3 of the ccRCC samples have an estimated tumour purity <60% and 1/3 have an estimated tumour purity >72%. ccRCC have a far lower median estimated tumour purity than papillary and chromophobe RCC samples (both P < 10^−16^, Wilcoxon rank-sum test). Clear cell RCC samples are still statistically significantly less pure than papillary and chromophobe RCC (P < 1 × 10^−11^) even after correcting for batch ID (Supplementary Table S1) and tissue source site (Supplementary Table S2) using linear mixed model analysis.

**Figure 2:**
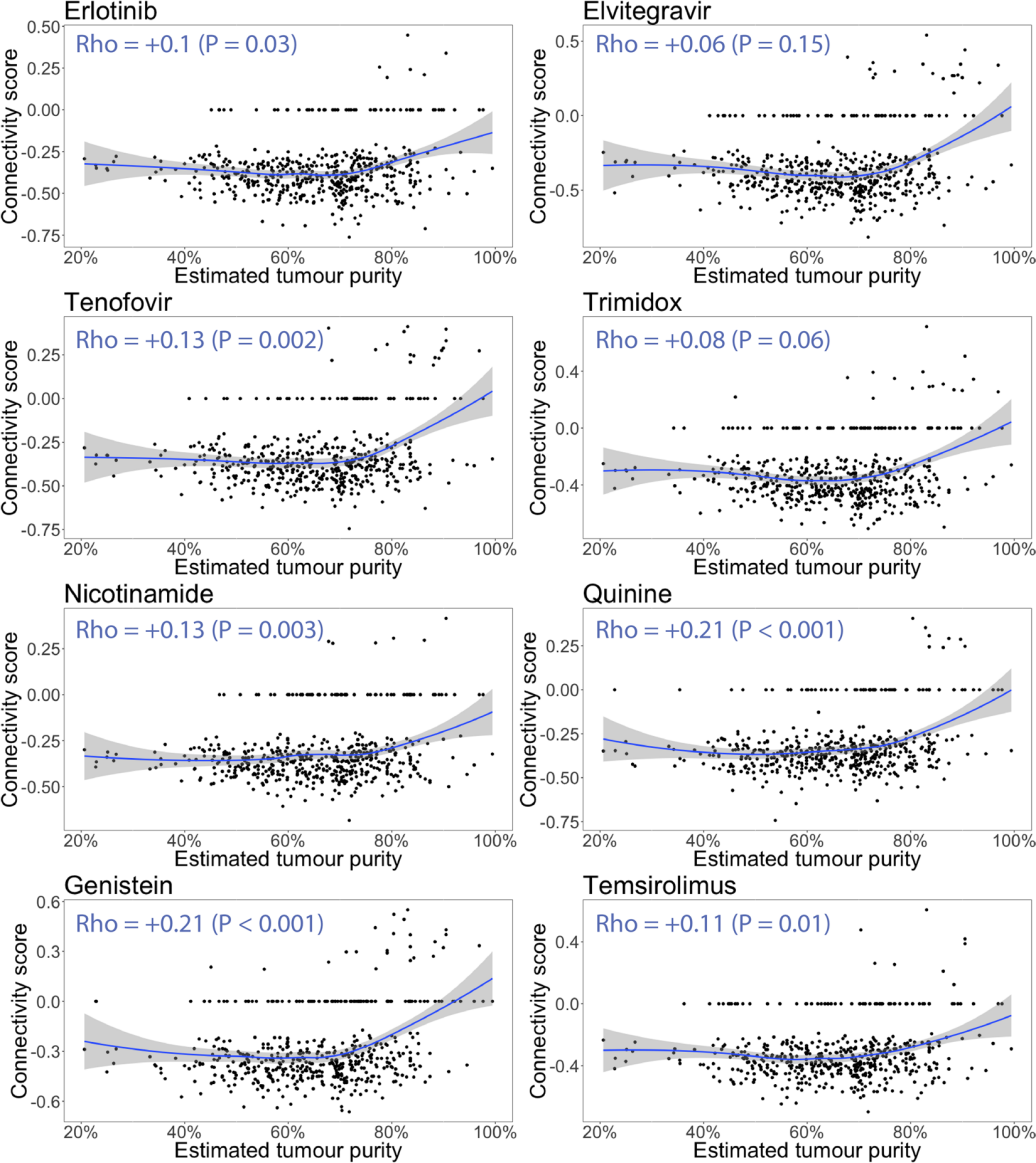
Connectivity scores of the top 8 drugs from previous analysis plotted against the estimated tumour purity. The blue line is a LOESS smoother. The Spearman rank correlation coefficients and P-values are displayed in the top left of each plot. With increasing estimated tumour purity, the connectivity scores start trending towards neutral (i.e. it suggests the drugs are expected to target the genes expressed by the non-cancerous cells present at lower tumour purity). When the correlation is tested using normal (i.e. Pearson) linear regression, or the samples <80% estimated tumour purity are compared to the samples >80% estimated tumour purity using the Wilcoxon rank-sum test, all resulting P values are below 0.001 (Supplementary Table S3).

The distribution of tumour purity estimations of all ccRCC samples (with estimated tumour purity and gene expression results available) has a mode of 70% and an interquartile range of 57–75% (Fig. 1). This figure further illustrates that compared to papillary and chromophobe renal cell carcinomas (RCC) from TCGA selected using the same criteria, ccRCC samples have both lower median estimated tumour purity and higher inter-sample variability in estimated tumour purity. Figure 2 displays the connectivity scores of each sample plotted against its estimated tumour purity. When the estimated tumour purity increases above 80%, the connectivity scores of 6 out of the top 8 drugs suddenly start trending towards neutral connectivity scores (P < 0.05, Spearman rank correlation). This result suggests the drugs are targeting the non-cancerous cells rather than ccRCC cells at lower tumour purity. Alternatively, excluding genes which become less differentially expressed with increasing tumour purity from the tumour sample signatures before calculating the connectivity scores would have led to the same conclusion, with the negative enrichment rate dropping from 25.6–47.4% to 0.4–16.4% (P < 10^−13^ for all 8 drugs, Supplementary Figs S1–S4).

**Figure 3:**
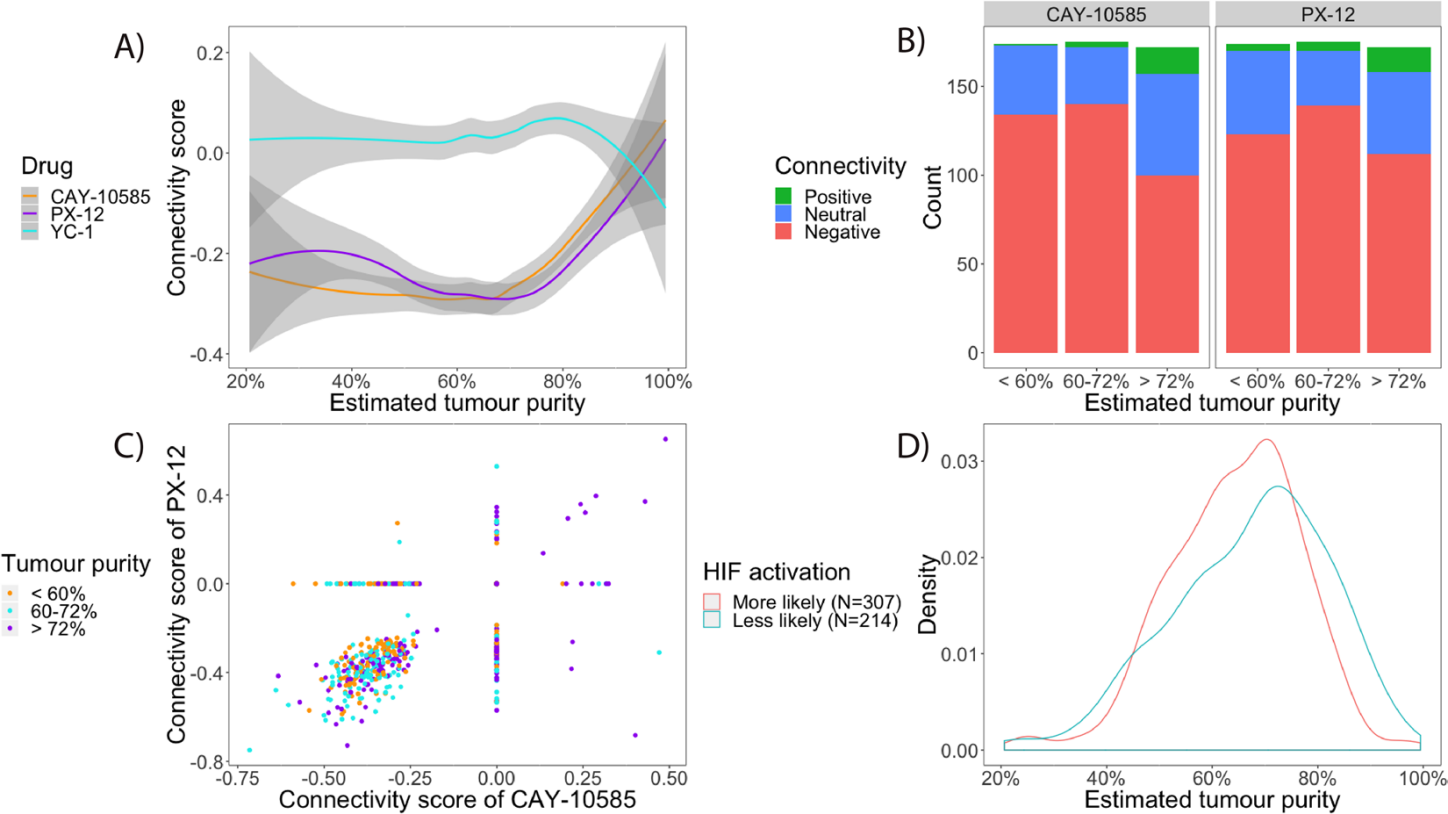
Relationship between connectivity score and estimated tumour purity for 3 drugs previously developed and/or identified as HIF-inhibitors. (**A**) Average connectivity score (LOESS smoother) versus estimated tumour purity. At <80% estimated tumour purity the connectivity scores for CAY-10585 and PX-12 remain consistently negative, whereas YC-1 remains neutral across the range. (**B**) The same data for CAY-10585 and PX-12 but plotted separately for each drug across each estimated tumour purity tertile. The increase in negative connectivity from <60% tumour purity to 60–72% tumour purity is positive but statistically non-significant for CAY-10585 (P = 0.2) and statistically significant for PX-12 (P < 0.001). However, the decrease in negative connectivity from 60–72% tumour purity to >72% tumour purity is highly statistically significant for both (P < 0.001). Associations were tested with Wilcoxon rank-sum test on the connectivity scores. (**C**) Connectivity scores of PX-12 plotted against the connectivity scores of CAY-10585. The connectivity scores of CAY-10585 and PX-12 correlate well (Spearman Rho = 0.56, P < 10^−16^). (**D**) Distribution of tumour purity for 2 groups with different probabilities of HIF-activation. Samples were put into the “More likely” group if it produced negative connectivity scores with both CAY-10585 and PX-12 (i.e. in the bottom left of (**C**), and into the “Less likely” group if otherwise. Samples in the “Less likely” HIF-activation group have a higher estimated tumour purity on average (P = 0.006, Wilcoxon rank-sum test). From this figure it is easy to see that at higher tumour purity (i.e. >80%) samples are relatively are increasingly less likely to show HIF-activation.

**Figure 4:**
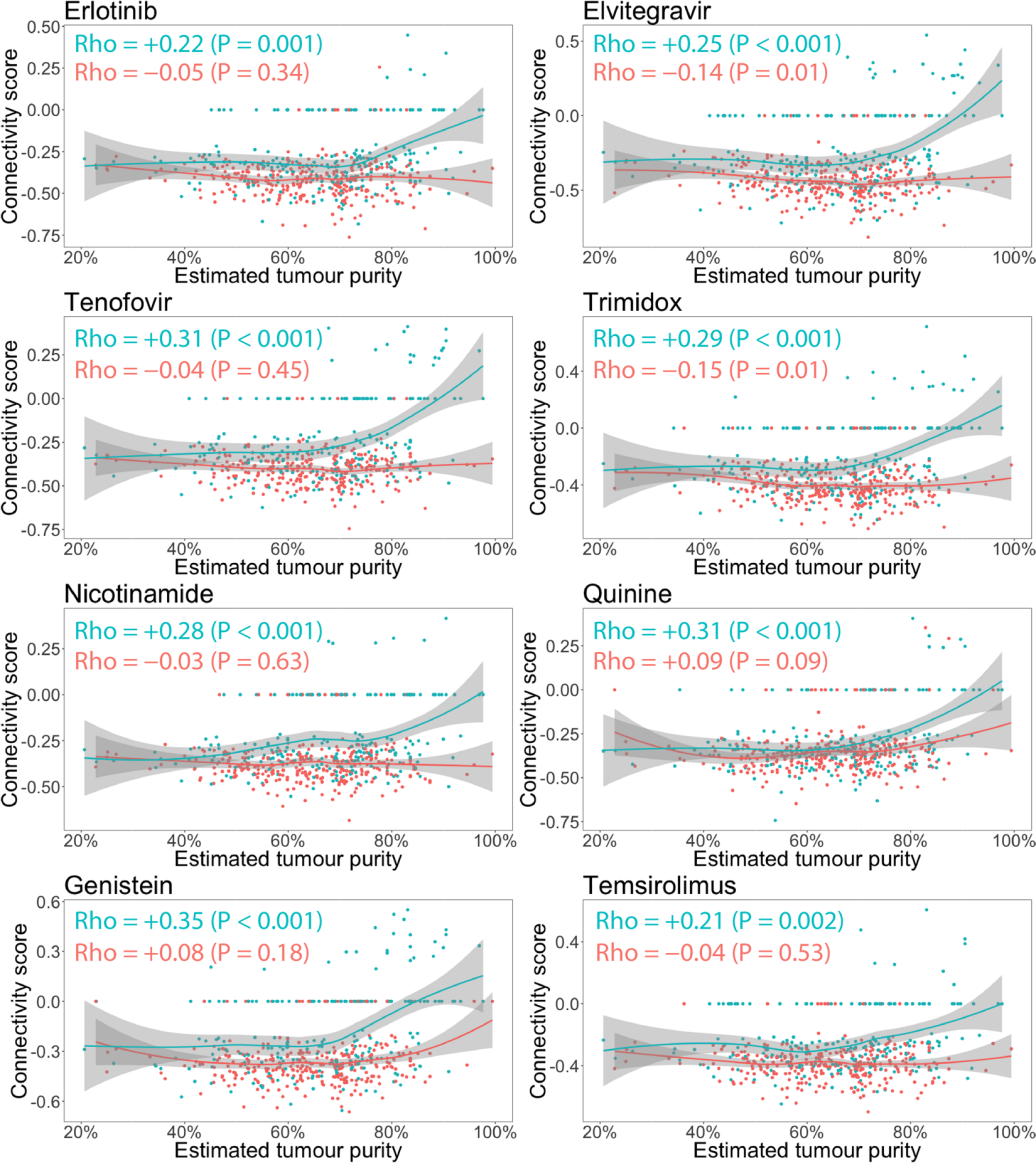
Same data, statistical tests and plots as Fig. 2, but now the samples have been colored according to the probability of HIF-activation. The red points correspond to the 307 samples in the “More likely” group, whereas the green points correspond to the 214 samples in the “Less likely” group. For samples with the higher probability of HIF-activation (red), the connectivity scores do not show a trend towards neutral with increasing tumour purity and even show the reverse trend for elvitegravir and trimidox. For samples with lower probability of HIF-activation, the trend of connectivity scores being neutralized with increasing estimated tumour purity is instead intensified.

**Figure 5:**
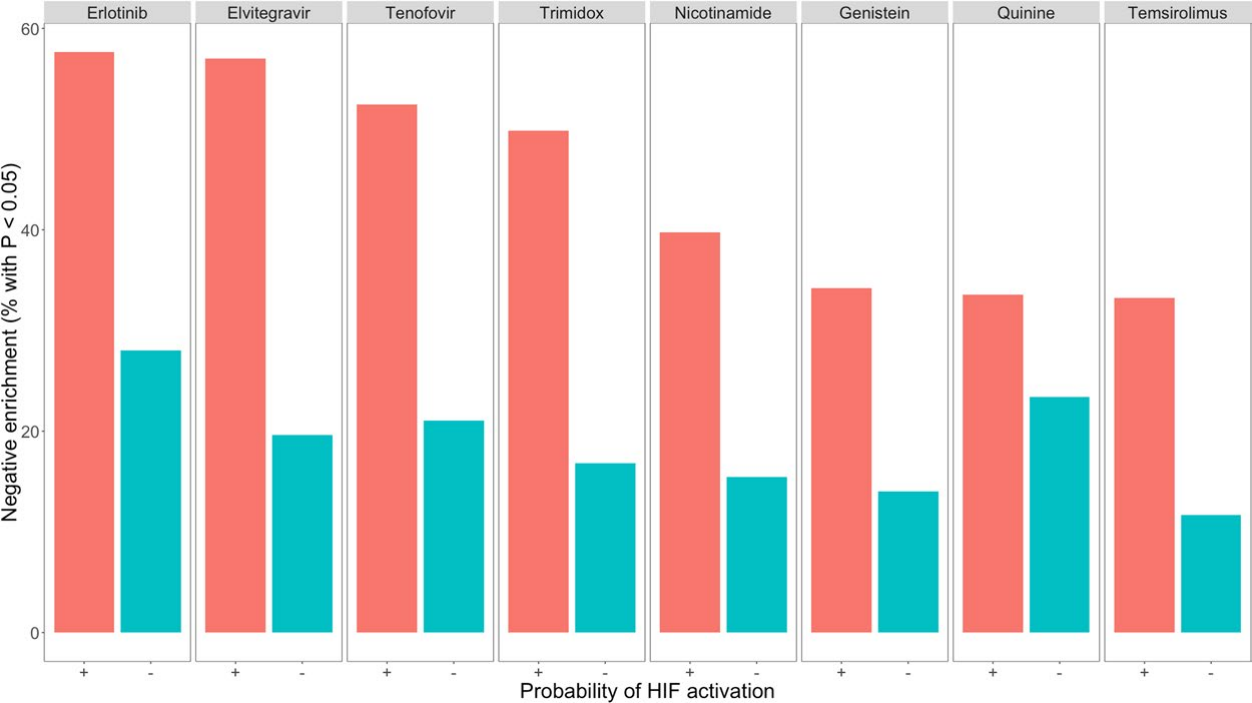
Frequency of statistically negative enrichment within samples more likely to show HIF-activation versus samples less likely to show HIF-activation. The difference was tested with a Chi-Square test on the raw counts. All frequency changes are highly statistically significant (P < 1 × 10^−6^) except for quinine (P = 0.02).

It is striking that all eight drugs (Fig. 2) show exactly the same trend towards neutrality with increasing tumour purity, including one of the current 2^nd^ line treatments (the mTOR inhibitor temsirolimus). Therefore, we investigated whether the same increase in connectivity scores occurred for inhibitors of the HIF-pathway, as activation of the HIF-pathway within the cancerous cells is a frequently observed hallmark of ccRCC^7^ (Fig. 3). Indeed, this proved to be the case for 2 of the 3 HIF-inhibitors (PX-12 and CAY-10585). This led to the idea to stratify the samples by probability of HIF-activation, which was deemed high if the tumour sample produced a negative connectivity score with both PX-12 and CAY-10585 and low if otherwise. Figure 4 illustrates that after stratification, the association between connectivity scores neutralizing with tumour purity completely disappeared and the global trend even reversed for elvitegravir and trimidox (P < 0.05, Spearman rank correlation). This finding suggests that HIF-activation is associated with both decreased tumour purity and (de)activation of pathways targeted by the drugs. The latter point is further illustrated by Fig. 5, in which samples with a higher probability of HIF-activation have doubled the rate of statistically significant negative connectivity scores (P < 1 × 10^−6^ for all 8 drugs except for quinine).

### Predictors of inter-sample variability in HIF-activation and estimated tumour purity

To determine which variables predict HIF-activation and/or the variation in estimated tumour purity, two separate linear regression analyses combined with backward elimination were performed. The input for these analyses were: VHL mutation/methylation status, tumour stage, mRNA subtype (as identified in the original TCGA analysis using hierarchical clustering analysis^9^), and the 3 most frequent chromosomal alterations observed in ccRCC: loss of chromosome 3p (i.a. containing the 4 most commonly mutated genes, i.e. VHL, PBRM1, BAP1 and SETD2^9^), gain of chromosome 5q (i.a. containing the SQSTM1 gene, increasing resistance to redox stress^10^) and loss of chromosome 14q (i.a. containing the HIF1A gene and the ASPP1 gene, a recently identified regulator of p53 activity^11^). Furthermore, the total number of chromosomal alterations (deletions of negative regulators and gains of positive regulators) in the pathway of genes that regulate p53 and the G1/S cell cycle transition were tested as potential predictors, as genes in this pathway are frequently chromosomally altered in ccRCC^12^. Batch ID, plate ID, ship date and tissue source site were included as random variables as these were considered potential sources of batch effects. The distribution of all included variables is listed in Table 1.

**Table 1:** Available data of included samples.

Variable name	Subcategories	Samples	% of total
VHL mutation/methylation status	No VHL mutation/methylation^Δ^	136	26.1
	VHL methylation	29	5.6
	Moderate impact VHL mutation	58	11.1
	High impact VHL mutation	102	19.6
	Missing*	196	37.6
Tumour stage	Stage I^Δ^	259	49.7
	Stage II	57	10.9
	Stage III	123	23.6
	Stage IV	79	15.2
	Missing^†^	3	0.6
mRNA subtype	m1^Δ^	143	27.4
	m2	88	16.9
	m3	93	17.9
	m4	85	16.3
	Missing*	112	21.5
Loss of chromosome 3p	No^Δ^	58	11.1
	Yes	455	87.3
	Missing^†^	8	1.5
Gain of chromosome 5q	No^Δ^	207	39.7
	Yes	306	58.7
	Missing^†^	8	1.5
Loss of chromosome 14q	No^Δ^	299	57.4
	Yes	214	41.1
	Missing^†^	8	1.5
Number of chromosomally altered genes which regulate p53 and the G1/S cell cycle transition	No subcategories (parameterized as a linear covariate)	513	98.5
	Missing^†^	8	1.5
Batch ID	15 unique batch ID’s	521	100%
Tissue source site	20 unique tissue source sites	521	100%
Plate ID^⋆^	18 unique plate ID’s	521	100%
Ship date^⋆^	15 unique ship dates	521	100%

510 out of the 521 samples (98%) with estimated tumour purity and gene expression data available were included in the linear regression analyses.

^Δ^Reference category.

^*^Missing value coded as separate category to avoid excluding samples.

^†^Samples with missing value excluded from subsequent analyses.

^⋆^Only used to predict the mean connectivity score of PX-12 and CAY-10585, as these batch variables are platform (i.e. RNA-seq, copy number, methylation) specific and tumour purity was estimated using the data from these 3 different platforms.

Given that missing mRNA subtype (N = 112) and missing VHL mutation/methylation data (N = 196) occurred frequently, missingness was used as a separate category. Otherwise, a complete case analysis was performed (Numbers missing: copy number data N = 8; tumour stage N = 3). The variable selection and estimation therefore took place on 510 out of the total 521 remaining ccRCC samples.

The 4 variables mRNA subtype, loss of chromosome 3p, gain of chromosome 5q and loss of chromosome 14q are significantly associated with the mean connectivity score of PX-12/CAY-10585 and estimated tumour purity (Adj. P value < 0.05 after performing univariate non-parametric tests and correcting for multiple testing using the Bonferroni method, Supplementary Tables S4 and S5). Furthermore, the number of chromosomally altered genes which regulate p53 and the G1/S cell cycle transition is associated with the mean connectivity score of PX-12/CAY-10585 (Adj. P value = 1 × 10^−9^) and tumour stage is associated with estimated tumour purity (Adj. P value = 0.007). As 4 out of the 7 variables are univariately associated with both outcomes, we performed stepwise linear regression using backward elimination, starting with all 7 variables including all possible sources of batch effects in both models (Supplementary Tables S6 and S7). For the final models presented in the main text we kept all variables which were selected at least >50% of the time after resampling the dataset with replacement and repeating the backward selection step (Supplementary Table S8). The results of the final models are presented in Table 2 (with the mean connectivity score of PX-12 and CAY-10585 as the dependent variable) and Table 3 (with estimated tumour purity as the dependent variable). To validate these findings, we performed repeated (10,000 times) 10-fold cross-validation which indeed further confirms that these results are robust and no substantial overfitting is taking place (Supplementary Figs S5 and S6).

**Table 2:** Coefficients final reduced linear model (R^2^ = 0.26) to explain the inter-sample variability in mean connectivity score of PX-12 and CAY-10585.

Coefficient	Estimate	Asymptotic P value^†^		Bootstrapped P value^△^	
Fixed effects					
Intercept	−0.29	1.45 × 10^−8^	***	<1 × 10^−4^	***
Estimated tumour purity	0.0015	0.01	*	0.01	*
mRNA subtype					
mRNA subtype m2	0.04	0.09		0.06	
mRNA subtype m3	0.04	0.12		0.10	
mRNA subtype m4	0.04	0.045	*	0.04	*
mRNA subtype missing	0.10	4 × 10^−6^	***	<1 × 10^−4^	***
Loss of chromosome 3p	−0.14	3.4 × 10^−9^	***	<1 × 10^−4^	***
Gain of chromosome 5q	−0.05	3 × 10^−4^	***	4 × 10^−4^	***
Loss of chromosome 14q	0.04	0.01	*	0.01	*
Number of chromosomal alterations in p53 and the G1/S cell cycle transition pathway (ranging from 0-15)	0.013	1.8 × 10^−9^	***	<1 × 10^−4^	***
Random effects					
Residual (SD)	0.15	—		—	

The coefficients of the full model are presented in Supplementary Table S6.

**Table 3:** Coefficients final reduced linear model (R^2^ = 0.19) to explain the inter-sample variability in estimated tumour purity.

Coefficient	Estimate	Asymptotic P value^†^		Bootstrapped P value^Δ^	
Fixed effects					
Intercept	76.1	<2 × 10^−16^	***	<1 × 10^−4^	***
Tumour stage					
-Stage II	1.6	0.38		0.37	
-Stage III	−2.6	0.06		0.06	
-Stage IV	0.5	0.76		0.76	
mRNA subtype					
-mRNA subtype m2	−3.3	0.05		0.06	
-mRNA subtype m3	−12.5	4 × 10^−13^	***	<1 × 10^−4^	***
-mRNA subtype m4	−3.8	0.03	*	0.01	*
-mRNA subtype missing	−0.1	0.95		0.93	
Loss of chromosome 3p	−4.2	0.02	*	0.07	
Gain of chromosome 5q	−2.5	0.03	*	0.03	*
Loss of chromosome 14q	−3.1	0.007	**	0.01	*
Random effects					
Residual (SD)	12.1	—		—	

The coefficients of the full model are presented in Supplementary Table S7.

^†^Calculated using T statistic.

^Δ^Determined resampling with replacement from the dataset and refitting model 10,000 times.

*P < 0.05; **<0.01; ***<0.001.

**Figure 6:**
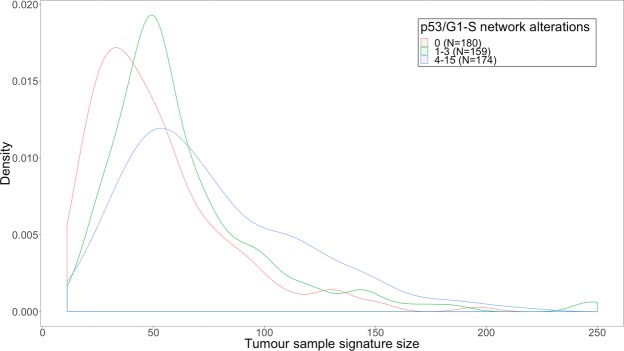
Density plot of the number of overlapping genes between tumour sample signatures and LINCS drug signatures, for the 3 tertiles of the number of genes chromosomally altered in genes that regulate p53 and the G1/S cell cycle transition. The number of genes increases from 0 alterations to 1–3 alterations (P = 9 × 10^−5^), from 1–3 alterations to 4–15 alterations (P = 6 × 10^−4^) and from 0 alterations to 4–15 alterations is even more significant (P = 1 × 10^−11^, all tested using the Wilcoxon rank-sum test).

**Figure 7:**
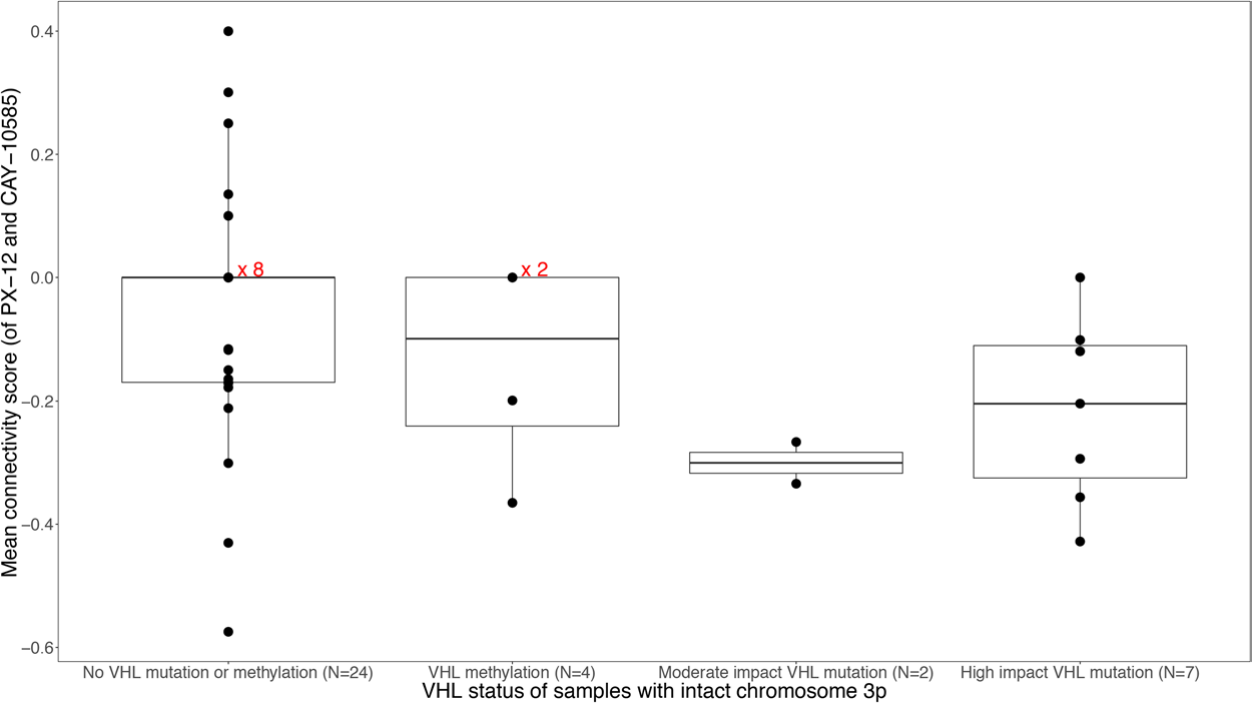
Mean connectivity score of PX-12 and CAY-10585 plotted against VHL status for the 37 samples which have not lost chromosome 3p and for which both mutation and methylation data is available. Sometimes points with connectivity score 0 are plotted over each other and given the number of samples to the right (i.e. x 2, x 8). VHL mutated or methylated samples (when the 3 types are grouped together) have statistically significantly lower connectivity scores than non-VHL mutated/methylated samples (P = 0.03, Wilcoxon rank-sum test).

Loss of chromosome 3p most strongly explains variability in the mean connectivity score of PX-12/CAY-10585: samples which lose this chromosome have a 0.14 points lower mean connectivity score on average (P = 3.4 × 10^−9^). Gain of chromosome 5q further decreases the mean connectivity score by an average of 0.05 points (P = 3 × 10^−4^). Samples with a missing mRNA subtype had on average a 0.1 points higher connectivity score (P = 4 × 10^−6^). In addition, each chromosomal alteration in p53 associated genes increases the mean connectivity score by an average of 0.013 points per alteration (P = 1.8 × 10^−9^). This is likely caused by the fact that the number of chromosomal alterations in p53 associated genes is strongly positively associated with the number of genes in the tumour sample signature (Fig. 6). These extra p53 associated genes effectively ‘dilute’ the HIF-associated genes in the tumour sample signature. Furthermore, note that although VHL mutation/methylation was not predictive when all samples were analysed together, it was predictive in the subset of 36 samples which did not lose chromosome 3p and which had mutation/methylation data available (Fig. 7).

With respect to tumour purity, the by far the strongest predictor of estimated tumour purity was mRNA subtype, with samples belonging to the m3 subtype being estimated to have an average 12.5% decrease compared to the m1 subtype (P = 4 × 10^−13^). The 2nd strongest effect size with regard to predicting estimated tumour purity was loss of chromosome 3p, estimated to decrease the estimated tumour purity by an average of 4.2% (P = 0.02). Loss of chromosome 14q (−2.5%, P = 0.03) and loss of chromosome 14q (−3.1%, P = 0.007) further contribute to decreases in estimated tumour purity.

The bootstrapped P values are generally more conservative, but except in the case of the association of 3p loss with estimated tumour purity, which produced bootstrapped P value of 0.07, the P values of all associations remain below 0.05. In summary, the 2 most frequent chromosomal alterations observed in ccRCC (3p loss and 5q gain, observed in 87% and 59% of samples respectively) are associated with both HIF activation (as evidenced by on average more negative connectivity scores with HIF inhibitors) and decreased estimated tumour purity regardless of analysis method (univariate non-parametric, multivariable regression using backward elimination and multivariable regression with all 7 preselected variables in the model including all possible sources of batch effects).

### Comparison with discordant methylation-clusters

In an earlier paper published in 2016, Winter *et al*.^13^ reported that the methylome of 25 ccRCC samples in the TCGA dataset clustered closer to papillary and chromophobe RCC samples than typical ccRCC samples. 24 of these samples (Table 4) were also included in our combined estimated tumour purity and gene expression analysis which allowed us to analyze the differences within these discordant samples and how they differ from samples with a typical ccRCC methylome. Samples are only compared to other samples for which Illumina HumanMethylation 450 BeadChip results are available (as other samples were not clustered in the methylation analysis). Of the samples with >80% estimated tumour purity, 15/45 (33%) were assigned to a discordant methylation cluster. Furthermore, the median estimated tumour purity of discordant samples is 17% higher (P = 2 × 10^−10^, Wilcoxon rank-sum test) and far more samples (15 out of 21, 71%) show no loss of chromosome 3p compared to samples with a typical ccRCC methylome (18/284, 6%), which is highly statistically significant (P < 1 × 10^−16^, Chi-squared test). Lastly, the 12 samples which upon histopathological re-evaluation were still considered ccRCC or unconventional ccRCC had lower median connectivity scores with PX-12 and CAY-10585 than the 12 samples deemed non-ccRCC (P = 0.03, Wilcoxon rank-sum test).

**Table 4:** ccRCC samples with methylation profiles which clustered with chromophobe or papillary RCC.

Sample prefix	Methylation cluster	Histopathological re-evaluation result	Estimated tumour purity	Mean connectivity score with PX-12 and CAY-10585	VHL mutation or methylation	mRNA subtype	Loss of 3p
TCGA-A3-3385	Papillary RCC	ccRCC	95.94	−0.36	High impact VHL mutation	m1	No
TCGA-CZ-5469	Papillary RCC	ccRCC	77.92	−0.22	No VHL mutation/methylation	m4	Yes
TCGA-B0-5098	Papillary RCC	unconventional-ccRCC	85.19	−0.11	No VHL mutation/methylation	Missing	Yes
TCGA-B0-5100	Papillary RCC	unconventional-ccRCC	79.56	−0.37	VHL methylation	m3	No
TCGA-B2-5636	Chromophobe RCC	unconventional-ccRCC	65.98	0.15	VHL methylation	m3	Missing
TCGA-B4-5378	Chromophobe RCC	unconventional-ccRCC	82.88	−0.39	Missing	Missing	No
TCGA-B8-4621	Papillary RCC	unconventional-ccRCC	70.24	0	No VHL mutation/methylation	m4	No
TCGA-B8-A54K	Chromophobe RCC	unconventional-ccRCC	85.54	0	VHL methylation	Missing	No
TCGA-BP-4760	Chromophobe RCC	unconventional-ccRCC	79.31	−0.20	VHL methylation	Missing	No
TCGA-DV-5567	Chromophobe RCC	unconventional-ccRCC	83.9	0	High impact VHL mutation	m3	No
TCGA-DV-5576	Chromophobe RCC	unconventional-ccRCC	77.64	−0.43	No VHL mutation/methylation	m3	No
TCGA-DV-A4VZ	Chromophobe RCC	unconventional-ccRCC	84.06	0	VHL methylation	Missing	No
TCGA-AK-3433	Chromophobe RCC	non-ccRCC	92.15	+0.14	Missing	Missing	Yes
TCGA-AK-3440	Chromophobe RCC	non-ccRCC	97.65	0	No VHL mutation/methylation	Missing	No
TCGA-AK-3453	Chromophobe RCC	non-ccRCC	99.47	−0.30	No VHL mutation/methylation	Missing	No
TCGA-B0-4688	Papillary RCC	non-ccRCC	72.77	0	Missing	Missing	No
TCGA-B0-4696	Chromophobe RCC	non-ccRCC	82.65	−0.12	Missing	Missing	Yes
TCGA-B0-4699	Chromophobe RCC	non-ccRCC	76.9	+0.29	Missing	Missing	No
TCGA-B0-5083	Chromophobe RCC	non-ccRCC	72.74	0	No VHL mutation/methylation	Missing	Missing
TCGA-B0-5117	Chromophobe RCC	non-ccRCC	88.39	0	No VHL mutation/methylation	Missing	No
TCGA-B0-5707	Papillary RCC	non-ccRCC	80.36	+0.15	No VHL mutation/methylation	Missing	Missing
TCGA-B8-5546	Papillary RCC	non-ccRCC	90.52	+0.40	No VHL mutation/methylation	m1	No
TCGA-CJ-5681	Papillary RCC	non-ccRCC	86.31	+0.16	No VHL mutation/methylation	m1	Yes
TCGA-EU-5907	Papillary RCC	non-ccRCC	91.83	−0.17	High impact VHL mutation	Missing	Yes

## Discussion

We found that HIF-activation likely acts as a confounder between estimated tumour purity and estimated drug repositioning potential of the previously reported top 8 drugs for ccRCC (Fig. 4). If instead of stratifying samples by probability of HIF-activation we had used the method of Aran *et al*.^4^, which automatically eliminates genes which are more strongly differentially expressed at lower estimated tumour purity, we would have incorrectly excluded genes representing a real biological signal.

To classify samples by probability of HIF-activation, we used the same assumptions underlying the computational drug repositioning method we used in the original analysis, namely that of gene expression signature reversal. This method predicts that drugs which downregulate genes upregulated by HIF-activation, and vice versa, are expected to produce negative connectivity scores with HIF-activated samples. This worked well for 2 out of 3 HIF-inhibitors (PX-12 and CAY-10585): the connectivity scores of tumour samples with these drugs produced concordant negative connectivity scores for the majority (58%) of samples. The third HIF-inhibitor (YC-1) did not correlate well with the other two or even produced more negative connectivity scores than expected by chance. This discrepancy is as of yet unexplained: YC-1 was tested in 13/14 of the same cell lines as CAY-10585 and in concentrations above the IC_50_^14^.

By studying predictors of the probability of HIF-activation in samples (with the mean connectivity score of PX-12 and CAY-10585 as the dependent variable), we learned that it can be attributed to two main underlying causes:

Firstly, observing HIF-activation depends on inactivation of the VHL gene due to loss of chromosome 3p (Table 2) or, in samples with an intact chromosome 3p, mutation or methylation of the VHL gene (Fig. 7). This result is in agreement with what is expected based on well-established pathophysiology^7^.

Secondly, observing HIF-activation can be interfered with by other factors, the most biologically plausible factor being deletions of negative regulators and gains of positive regulators in the genes that regulate p53 and the G1/S cell cycle transition. These copy number alterations result in many more genes differentially expressed in the tumour samples (Fig. 6), which subsequently decrease the likelihood of observing HIF-activation (Table 2). This can be explained by the way connectivity scores are calculated^15^. When genes which are not associated with HIF expression are added to the tumour sample signature, on average the genes rank closer to the middle when they are compared to the genes ordered by changes in expression after administration of HIF-inhibitors. Statistical procedures which only look at the overlap of genes differentially expressed between the tumour sample signature and drug signature may be less sensitive to this effect^16^. Missing mRNA subtype was also found to be an important negative predictor of HIF-activation, for which we have not found a good explanation yet.

When we explored possible predictors of estimated tumour purity, we found that mRNA subtype (as identified in the original TCGA analysis^9^) produced the largest contribution. Compared to the m1 subtype, samples belonging to the m3 subtype have on average a 12.5% lower estimated tumour purity (Table 3). Perhaps not coincidentally, patients with the m3 subtype also experience the worst overall survival^9^. After mRNA subtype, loss of chromosome 3p, associated with increased HIF-activation, had the strongest single effect of any variable (4.2% reduction in estimated tumour purity).

When we compared our gene expression-based results to the methylome clustering results produced by Winter *et al*.^13^, we found that the 24 samples with papillary-like and chromophobe-like methylomes, similar to samples with lower probability of HIF-activation, had a higher median estimated tumour purity as well as a lower rate of chromosome 3p loss. This therefore offers a complementary/alternative explanation for the outlying results observed at the high (>80%) estimated tumour purity of the scale. However, as the 12 samples which were independently histopathologically reclassified as conventional ccRCC (N = 2) or unconventional ccRCC (N = 10) had lower median connectivity scores with the HIF-inhibitors than the 12 samples reclassified as chromophobe or papillary RCC, observed HIF-activation (as a correlate of ccRCC-likeness) does seem to correlate better with independent histopathological review than methylation clustering alone.

For both models presented in Tables 2 and 3 it seems plausible that the inter-sample variation can be explained by real biology, rather than by random technical variation (e.g. variation in sampling or processing of the tissue) as including all possible sources of batch effects (the full linear mixed model outputs presented in the Supplementary Tables S6 and S7) does not affect the direction or statistical significance of any of the variables of interest. It is of course possible that any hidden batch effects are still in the data, but principal component analysis of the gene expression data did not show any clustering to suggest any hidden batch effects^2,9^.

It is biologically plausible that the cancer cells behave differently (and thus gene expression within tumour cells changes) if the tumour microenvironment becomes purer or less pure (e.g. as a reaction to the invasion of immune cells or infiltration of blood vessels). However, the bulk RNA-seq data from TCGA do not allow us to distinguish directly which mRNA is produced by cancer cells and which is produced by non-cancerous cells. We therefore heavily relied on previously established knowledge to interpret our findings (i.e. HIF-activation causes lower tumour purity due to increased vascularity, instead of decreased tumour purity causing HIF-activation). To conclusively answer this question and explore new hypotheses, it would require a large single cell RNA-seq dataset of the ccRCC tumour micro-environment from many different patients, so that the impact of variation in cancer cell genetic identity (i.e. mutations, copy number alterations, methylation) can be distinguished from variation in cell type composition of the micro-environment. However, because of the expense and newness of single cell RNA-seq no such dataset exists yet (to our knowledge), and only the TCGA dataset contains enough samples (500+ ccRCC and 10,000+ of all tumour types combined) to really quantify the impact of inter-sample variation in gene expression and relate this to other genomic determinants.

In conclusion, we demonstrated using the example of HIF-activation that confounding between tumour purity and gene expression can occur. Therefore, we recommend correcting for tumour purity in bulk RNA-seq differential expression analyses only after any other possible biological causes are included in the model.

## Materials and Methods

All data was processed with R version 3.4.0 and R/Bioconductor packages. All tests produced two-sided P-values and were considered statistically significant at P < 0.05.

### Connectivity scores versus estimated tumour purity

The tumour sample and drug signatures were calculated as described previously^2^. Briefly, the tumour sample signatures were generated by comparing 538 individual tumour gene expression profiles to 72 normal kidney tissue gene expression profiles, both generated by TCGA^9^. After filtering out genes with low expression (counts per million <0.5) and applying the Benjamini-Hochberg procedure to calculate the False Discovery Rate (FDR), genes with an FDR <1% were included in the tumour sample signatures. As an additional quality control filter, only samples which passed the independent quality control check of the Toil recompute effort (a recent project to reanalyze all gene expression results in an updated and uniform way) were included^8^. Drug signatures, based on data generated by The Library of Integrated Network-Based Cellular Signatures (LINCS) Program^17^, were calculated with the goal of approximating the effect in the average tumour cell line. This was accomplished through the use of a linear model with drug concentration as a linear parameter, and cell line, exposure duration, and batch as categorical variables.

To estimate tumour sample purity, we used the previously published consensus measurements of tumour purity estimations (CPE)^4^. In summary, CPE is based on 3 different data types and algorithms: copy number data (ABSOLUTE), methylation data (LUMP) and transcriptomic data (ESTIMATE). For simplicity and clarity’s sake, CPE is hereafter simply referred to as “Estimated tumour purity”. TCGA batch information (Batch ID’s, Plate ID’s, Ship dates and Tissue source sites) were downloaded using the TCGA Batch Effects Tool^18^ (version 2.0).

Connectivity scores (the method used to compare the drug and tumour signatures) were calculated using the PharmacoGx package^16^ (version 1.8.3). A negative connectivity score implies that the drug is expected to (partially) reverse the tumour signature. The correlation between the connectivity scores and estimated tumour purity was tested non-parametrically using the Spearman rank correlation test. A positive association (i.e. more positive connectivity scores at higher tumour purity %) suggests that the gene expression reversal occurs mainly in non-cancerous cells. Conversely, a negative association implies the opposite, namely that as the % of tumour material in a sample increases so does the drug repositioning potential, which suggests that the drug is targeting the tumour cells.

Genes were classified as more differentially expressed with increasing tumour purity if upregulated and downregulated genes showed a statistically significant (P < 0.05) positive, respectively negative Spearman rank correlation with increasing tumour purity. If it showed the opposite relationship, the gene was classified as becoming less differentially expressed with increasing tumour purity. Lastly, if the Spearman rank correlation with estimated tumour purity was statistically insignificant (P > 0.05) regardless of direction, the gene was classified as insensitive to increasing tumour purity.

### Predictors of inter-sample variability in HIF-activation and estimated tumour purity

Mutation and methylation data of tumour samples were downloaded using the TCGAbiolinks package (version 2.6.12)^19^. Only mutations and methylation of the VHL gene were considered because this gene is considered the key trigger in starting the angiogenesis cascade. VHL was considered methylated if the beta value for probe cg15267345 exceeded 0.2 (as in previous methylation analyses^9,20–22^). The copy number data processed by GISTIC (an algorithm used to call chromosomal alterations, i.e. deletions or gains) was downloaded using the RTCGAToolbox package (version 2.8.0)^23^. For tumour samples analysed in duplicate, the average value of the variable was used in the analysis.

The following variables were investigated for their association with observed HIF activation (for which we used negative connectivity scores with the HIF inhibitors PX-12 and CAY-10585 as a proxy) and estimated tumour purity: Estimated tumour purity, VHL mutation/methylation status, tumour stage, mRNA subtype, loss of chromosome 3p, gain of chromosome 5q, loss of chromosome 14q and number of chromosomal alterations in the p53 and the G1/S cell cycle transition pathway. Selected variables were univariately associated with HIF-activation and estimated tumour purity using non-parametric statistical tests: the Wilcoxon Rank-sum test for categorical variables with 2 groups (e.g. no 3p loss versus 3p loss), the Kruskal-Wallis test for variables with more than 2 groups (e.g. mRNA subtype) and Spearman’s rank correlation coefficient for continuous variables (e.g. Number of chromosomally altered genes which regulate p53 and the G1/S cell cycle transition). To explain inter-sample variability in HIF-activation and estimated tumour purity, linear mixed models with all variables included were fitted. Batch ID, plate ID, tissue source site and ship date were parameterized as random effects. Estimated tumour purity, plate ID and ship date were only included as a variable to predict inter-sample variability in HIF-activation because estimated tumour purity can’t be used to predict itself and plate ID + ship date are specific only to the RNA-seq experiments. Variables were sequentially dropped from the model until only variables with P values lower than 0.05 were left. This process was repeated on 1,000 bootstrapped datasets (sampled with replacement). Variables which remained in the model after backward selection at least 50% of the time were kept in the final 2 models presented in the main article. The bootstrapped P values of the final models were determined by sampling the original dataset with replacement 10,000 times. Lastly, for each final model, repeated (10,000 times) 10-fold cross-validation was performed to detect possible overfitting.

### Comparison with discordant methylation-clusters

In a previously published methylation analysis, Winter *et al*. analysed 319 TCGA samples for which Illumina HumanMethylation 450 BeadChip results were available and concluded that based on the methylation patterns, 15 of the ccRCC samples showed closer similarity to chromophobe RCC and 10 to papillary RCC^13^. Additionally, they asked 2 independent pathologists with expertise in renal tumour pathology to re-evaluate the ccRCC samples based on publicly available TCGA diagnostic and histological slide images. The discordant samples and re-evaluation results were extracted from Supplementary Table S1 and joined with our own gene-expression based results and tumour purity estimations.

## Supplementary information

Supplementary material

## Data Availability

The R code, additional input files needed and the resulting datasets generated during the current study are included in the public GitLab repository (https://gitlab.com/k.k.m.koudijs/personalised_DR_ccRCC).
