# Supplementary material for: The impact of estimated tumour purity on gene expression-based drug repositioning of Clear Cell Renal Cell Carcinoma samples
Source: Sci Rep. 2019 Feb 21;9:2495. doi: 10.1038/s41598-019-39891-y (PMC6385172; doi:10.1038/s41598-019-39891-y)
Supplement: Supplementary file 1 — Supplementary material [file 41598_2019_39891_MOESM1_ESM.pdf]

The impact of estimated tumour purity on gene expression-based drug repositioning of Clear Cell Renal Cell Carcinoma samples

Supplementary material

Karel K.M. Koudijs[1], Anton G.T. Terwisscha van Scheltinga[1], Stefan Böhringer[2], Kirsten J.M. Schimmel[1], Henk-Jan Guchelaar[1*]

1. Department of Clinical Pharmacy & Toxicology, Leiden University Medical Centre, Leiden, the Netherlands.
2. Department of Medical Statistics, Leiden University Medical Centre, Leiden, the Netherlands.

* = corresponding author

Table of contents:

Item	Description	Page
Supplementary Figure S1	Each gene shared in common between the LINCS array and the set of genes sufficiently expressed in the tumour samples categorized by its association with estimated tumour purity.	3
Supplementary Figure S2	Number of genes included in the tumour sample signatures before and after excluding genes which become less differentially expressed with increasing tumour purity.	3
Supplementary Figure S3	Connectivity scores of tumour samples with the previously identified top 8 drugs before and after excluding genes which become less differentially expressed with increasing tumour purity.	4
Supplementary Figure S4	Statistically significant negative enrichment rate of tumour samples with the previously identified top 8 drugs before and after excluding genes which become less differentially expressed with increasing tumour purity.	4
Supplementary Figure S5	Mean squared errors (MSE) resulting from repeated (10,000 times) 10-fold cross-fold validation of the models to explain the variability in mean connectivity score of PX-12 and CAY-10585.	5
Supplementary Figure S6	Mean squared errors (MSE) resulting from repeated (10,000 times) 10-fold cross-fold validation of the models to explain the variability in estimated tumour purity.	5
Supplementary Table S1	Coefficients linear mixed model analysis of estimated tumour purity (in %) across the 3 types of renal cell carcinomas with batch ID included as random effect.	6
Supplementary Table S2	Coefficients linear mixed model analysis of estimated tumour purity (in %) across the 3 types of renal cell carcinomas with tissue source site included as random effect.	6
Supplementary Table S3	Alternative methods used to calculate P values of association between connectivity scores of drugs and estimated tumour purity.	6
Supplementary Table S4	Non-parametric test results of univariate associations between the mean connectivity score of PX-12 and CAY-10585 and selected covariates.	7
Supplementary Table S5	Non-parametric test results of univariate associations between estimated tumour purity and selected covariates.	7
Supplementary Table S6	Coefficients full linear mixed model (12 tested variables) to explain the variability in mean connectivity score of PX-12 and CAY-10585. Counterpart to the reduced model presented in table 2.	8
Supplementary Table S7	Coefficients full linear mixed model (9 tested variables) to explain the variability in estimated tumour purity. Counterpart to the reduced model presented in table 3.	9
Supplementary Table S8	Frequency of selected model variables after resampling dataset with replacement for 1,000 times and backward regression using P < 0.05.	10

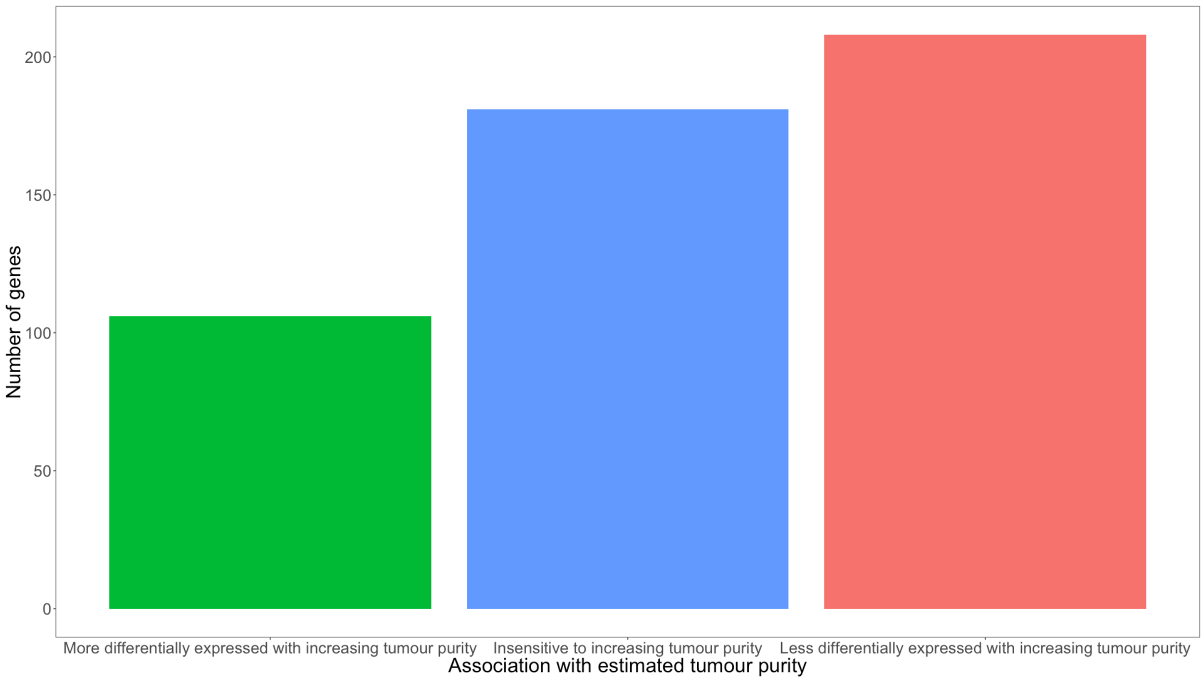

Supplementary Figure S1: Each gene shared in common between the LINCS array and the set of genes sufficiently expressed in the tumour samples categorized by its association with estimated tumour purity. Almost half (42%) of genes become less differentially expressed with increasing tumour purity.

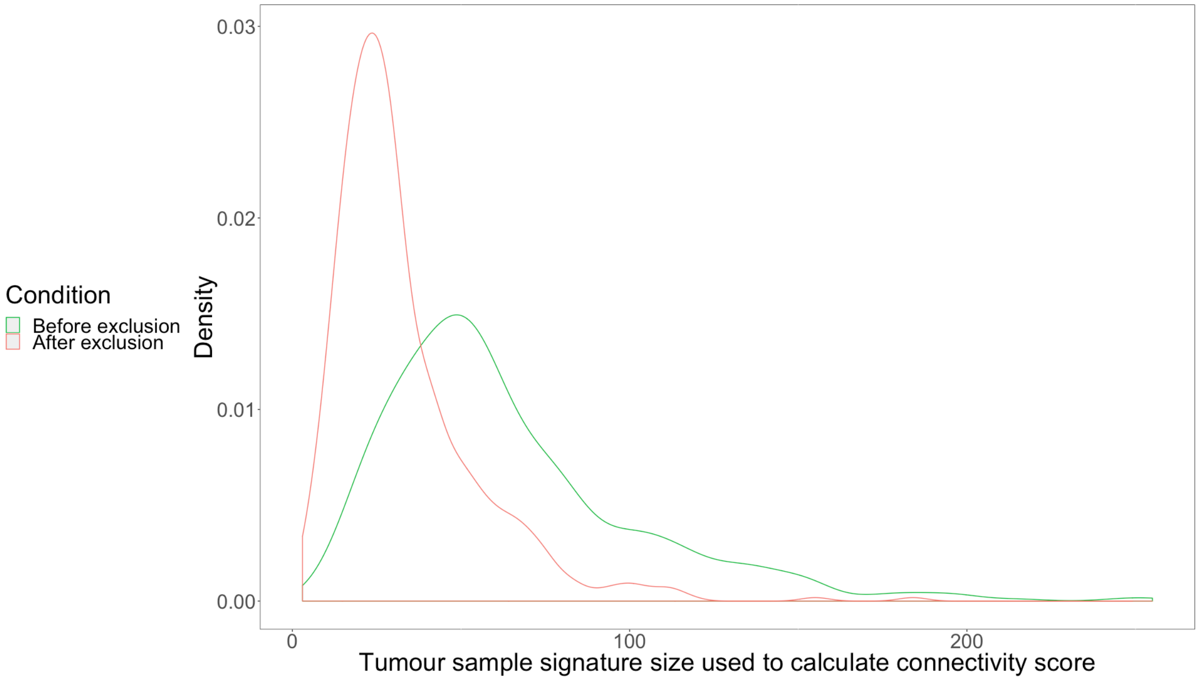

Supplementary Figure S2: Number of genes included in the tumour sample signatures before and after excluding genes classified as becoming less differentially expressed with increasing tumour purity. The median tumour sample signature contains 54 genes before excluding genes versus 27 genes after excluding genes. 500 tumour samples have at least 10 genes remaining in both types of tumour signatures and were used to create Supplementary Fig. S3-S4.

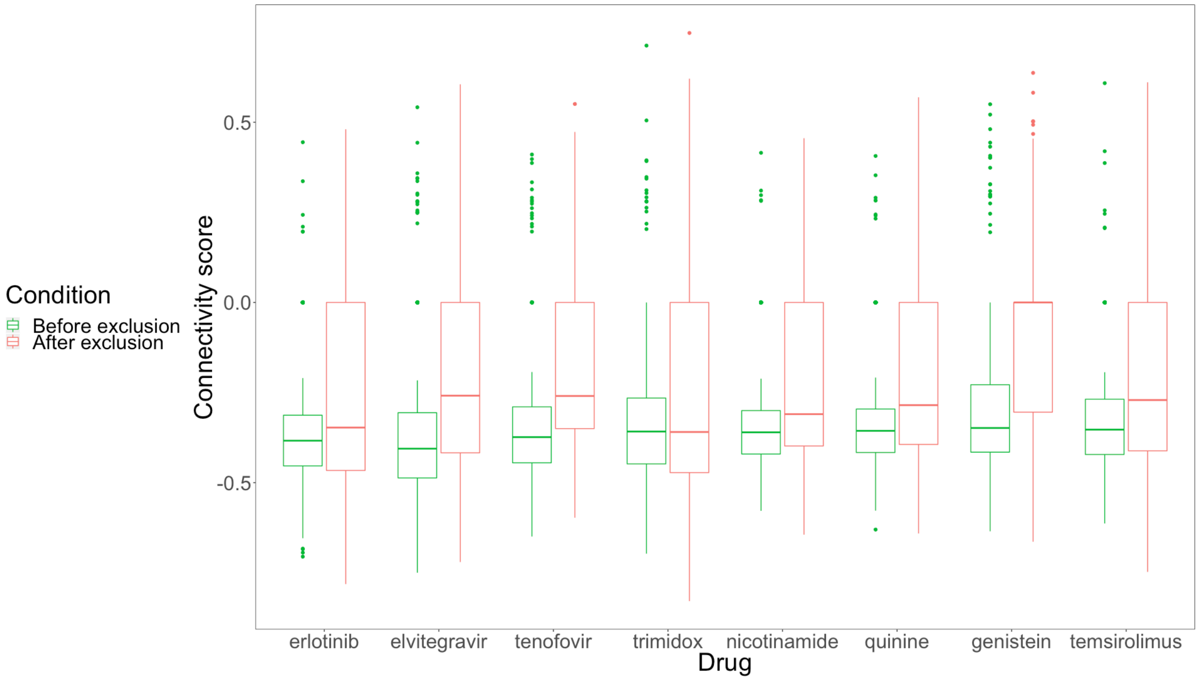

Supplementary Figure S3: Connectivity scores of tumour samples with the previously identified top 8 drugs before and after excluding genes which become less differentially expressed with increasing tumour purity. Except for trimidox, all connectivity scores become significantly more neutral after excluding the genes (P < 0.001 for each drug, Wilcoxon rank-sum test).

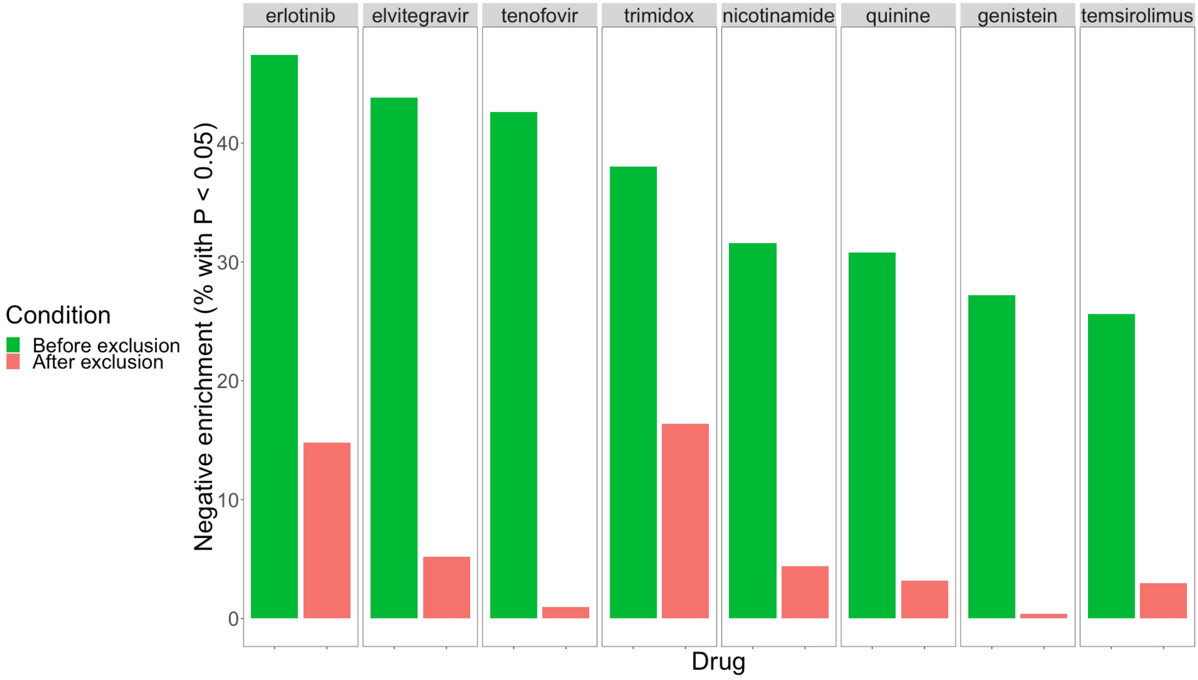

Supplementary Figure S4: Statistically significant negative enrichment rate of tumour samples with the previously identified top 8 drugs before and after excluding genes which become less differentially expressed with increasing tumour purity. The negative enrichment rate drops from 25.6-47.4% to 0.4-16.4% ($P < 10^{-13}$ for each drug, Chi-square test on the counts).

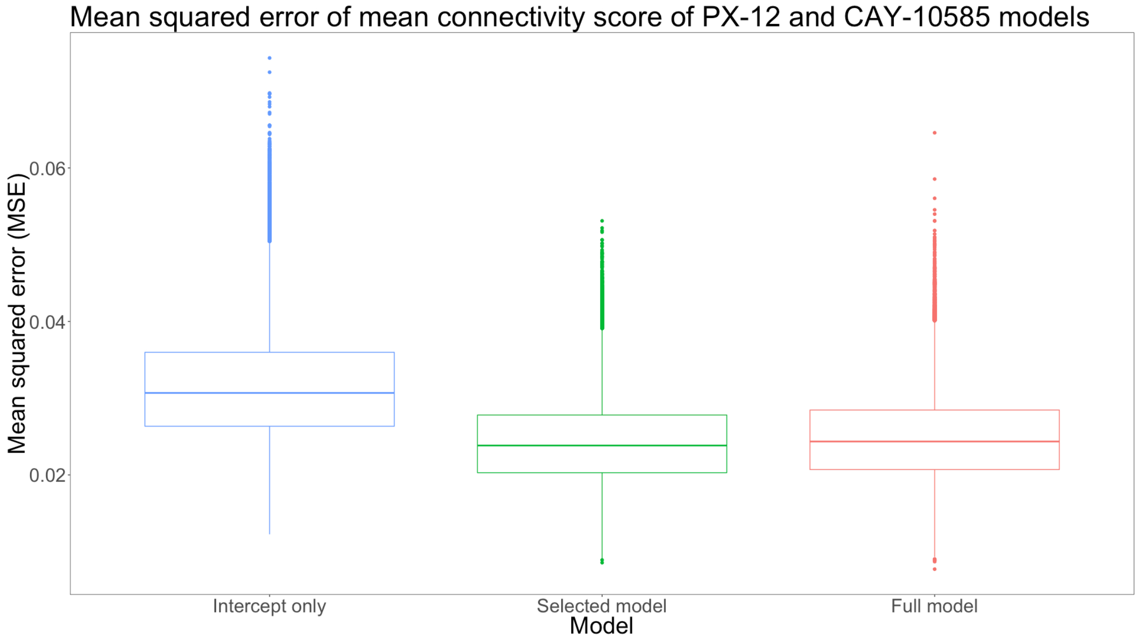

Supplementary Figure S5: Mean squared errors (MSE) resulting from repeated (10,000 times) 10-fold cross-fold validation of the models to explain the variability in mean connectivity score of PX-12 and CAY-10585. The median MSE for the intercept only model is 0.0307, for the selected model 0.0238 (22% reduction versus intercept only model, $P < 10^{-16}$ Wilcoxon Rank-Sum test) and for the full model with all variables 0.0243 (2% increase over selected model, $P < 10^{-16}$ Wilcoxon Rank-Sum test).

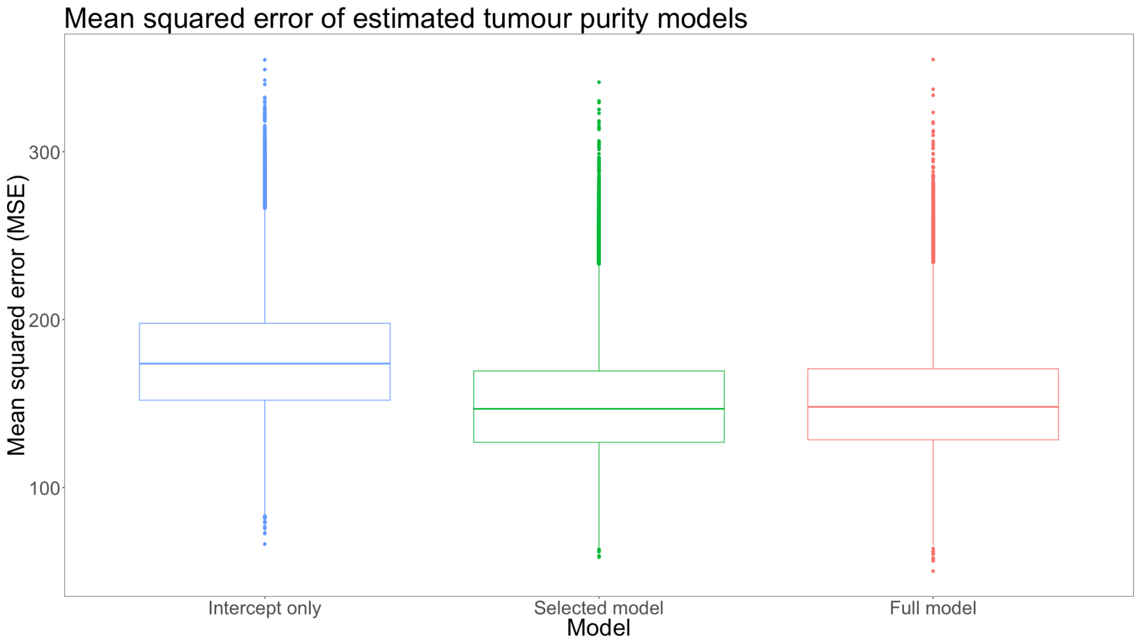

Supplementary Figure S6: Mean squared errors (MSE) resulting from repeated (10,000 times) 10-fold cross-fold validation of the models to explain the variability in estimated tumour purity. The median MSE for the intercept only model is 173.8, for the selected model 146.9 (15.5% reduction versus intercept only model, $P < 10^{-16}$ Wilcoxon Rank-Sum test) and for the full model with all variables 0.0243 (0.7% increase over selected model, $P = 2 \times 10^{-6}$ Wilcoxon Rank-Sum test).

Supplementary Table S1: Coefficients linear mixed model analysis of estimated tumour purity (in %) across the 3 types of RCC with batch ID included as random effect.

Coefficient	Estimate	Standard error	P value†	
**Fixed effects**				
Intercept△	65.7	0.70		
Tumour type			1.3×10^{-14}	***
- Papillary RCC	14.8	1.12		
- Chromophobe RCC	22.2	2.29		
**Random effects**				
Batch ID (SD)	1.5		0.14	
Residual (SD)	12.9		-	

Legend:

- *△: Intercept should be interpreted as the mean estimated tumour purity of clear cell RCC.*
- *†: Calculated using the Likelihood Ratio Test between model with versus without variable (e.g. with and without tumour type)*
- ****: P < 0.001.*

Supplementary Table S2: Coefficients linear mixed model analysis of estimated tumour purity (in %) across the 3 types RCC with tissue source site included as random effect.

Coefficient	Estimate	Standard error	P value†	
**Fixed effects**				
Intercept△	67.1	0.985		
Tumour type			3.4×10^{-11}	***
- Papillary RCC	13.0	1.362		
- Chromophobe RCC	20.7	2.278		
**Random effects**				
Tissue source site (SD)	2.5	-	0.003	**
Residual (SD)	12.8	-	-	

Legend:

- *△: Intercept should be interpreted as the mean estimated tumour purity of clear cell RCC.*
- *†: Calculated using the Likelihood Ratio Test between model with versus without variable (e.g. with and without tumour type*
- ***: P < 0.01; ***: P < 0.001.*

Supplementary Table S3: Alternative methods used to calculate P values of association between connectivity scores of drugs and estimated tumour purity.

Drug	P value of correlation tested using normal (i.e. Pearson) linear regression		P value Wilcoxon rank sum test (< 80% vs > 80% purity)	
Erlotinib	0.0004	***	0.0004	***
Elvitegravir	0.0002	***	3×10^{-6}	***
Tenofovir	3.3×10^{-8}	***	3×10^{-6}	***
Trimidox	9.9×10^{-5}	***	4×10^{-5}	***
Nicotinamide	4.4×10^{-6}	***	0.0008	***
Quinine	9.5×10^{-10}	***	1.7×10^{-6}	***
Genistein	4.9×10^{-10}	***	6.4×10^{-7}	***
Temsirolimus	0.0009	***	0.002	***

*Legend: ***: P < 0.001.*

Supplementary Table S4: Non-parametric test results of univariate associations between the mean connectivity score of PX-12 and CAY-10585 and selected covariates.

Variable name	Descriptive statistics (*m* = median)	P value	Adj. P value †	
VHL mutation/methylation status	No VHL mutation or methylation (*m*: −0.3)<br>VHL methylation (*m*: −0.28)<br>Moderate impact VHL mutation (*m*: −0.33)<br>High impact VHL mutation (*m*: −0.3)<br>Missing (*m*: −0.31)	0.52	1	
Tumour stage	Stage I: (*m*: −0.31),<br>Stage II (*m*: −0.31),<br>Stage III (*m*: −0.31),<br>Stage IV (*m*: −0.23)	0.03	0.19	
mRNA subtype	m1 (*m*: −0.37)<br>m2 (*m*: −0.31)<br>m3 (*m*: −0.3)<br>m4 (*m*: −0.29)<br>Missing (*m*: −0.15)	8.4×10^{-10}	5.9×10^{-9}	***
Loss of chromosome 3p	No (*m*: −0.12), Yes (*m*: −0.31)	3.8×10^{-9}	2.7×10^{-8}	***
Gain of chromosome 5q	No (*m*: −0.27), Yes (*m*: −0.31)	1.5×10^{-3}	0.01	*
Loss of chromosome 14q	No (*m*: −0.32), Yes (*m*: −0.28)	5.0×10^{-3}	0.04	*
Number of chromosomally altered genes which regulate p53 and the G1/S cell cycle transition	Rho = +0.28<br>(Bootstrapped 95% confidence interval ranges from 0.19 to 0.36)	1.4×10^{-10}	1×10^{-9}	***

Legend:

- *†: P value was adjusted using Bonferroni correction (i.e. multiplied by 7)*
- **: Adj. P < 0.05; **: Adj. P < 0.01; ***: Adj. P < 0.001.*

Supplementary Table S5: Non-parametric test results of univariate associations between estimated tumour purity and selected covariates.

Variable name	Descriptive statistics (*m* = median)	P value	Adj. P value†	
VHL mutation/methylation status	No VHL mutation or methylation (*m*: 69.3)<br>VHL methylation (*m*: 68.8)<br>Moderate impact VHL mutation (*m*: 63.6)<br>High impact VHL mutation (*m*: 69.0)<br>Missing (*m*: 66.5)	0.07	0.52	
Tumour stage	Stage I: (*m*: 69.1)<br>Stage II (*m*: 68.9)<br>Stage III (*m*: 60.5)<br>Stage IV (*m*: 66.7)	0.001	0.007	**
mRNA subtype	m1 (*m*: 70.3)<br>m2 (*m*: 67.3)<br>m3 (*m*: 55.9)<br>m4 (*m*: 65.0)<br>Missing (*m*: 71.6)	1.5×10^{-12}	1×10^{-11}	***
Loss of chromosome 3p	No (*m*: 76.9), Yes (*m*: 66.4)	1.6×10^{-4}	0.001	**
Gain of chromosome 5q	No (*m*: 69.6), Yes (*m*: 64.7)	9.3×10^{-4}	0.007	**
Loss of chromosome 14q	No (*m*: 69.4), Yes (*m*: 62.8)	2.1×10^{-6}	1.4×10^{-5}	***
Number of chromosomally altered genes which regulate p53 and the G1/S cell cycle transition	Rho = −0.09<br>(Bootstrapped 95% confidence interval ranges from −0.17 to 0.0)	0.05	0.37	

Legend:

- *†: P value was adjusted using Bonferroni correction (i.e. multiplied by 7)*
- **: Adj. P < 0.05; **: Adj. P < 0.01; ***: Adj. P < 0.001.*

Supplementary Table S6: Coefficients full linear mixed model (12 tested variables) to explain the variability in mean connectivity score of PX-12 and CAY-10585. Counterpart to the reduced model presented in table 2.

Coefficient	Estimate	Standard error	P value†	
**Fixed effects**				
Intercept	-0.2875	0.0523	-	
Estimated tumour purity	0.0013	0.0006	0.02	*
VHL mutation/methylation status			0.92	
- VHL methylation	0.0112	0.0322		
- Moderate impact VHL mutation	-0.0079	0.0240		
- High impact VHL mutation	0.0136	0.0205		
- Missing	0.0081	0.0205		
Tumour stage			0.21	
- Stage II	-0.0073	0.0226		
- Stage III	-0.0220	0.0180		
- Stage IV	0.0241	0.0215		
mRNA subtype			0.0004	***
- mRNA subtype m2	0.0343	0.0215		
- mRNA subtype m3	0.0414	0.0226		
- mRNA subtype m4	0.0436	0.0219		
- mRNA subtype missing	0.0974	0.0215		
Loss of chromosome 3p	-0.1286	0.0228	3×10^{-8}	***
Gain of chromosome 5q	-0.0542	0.0144	2×10^{-4}	***
Loss of chromosome 14q	0.0376	0.0149	0.01	*
Number of chromosomal alterations in p53 and the G1/S cell cycle transition pathway (ranging from 0-15)	0.0126	0.0021	3×10^{-9}	***
**Random effects**				
Batch ID (SD)	0.0000	-	1	
Plate ID (SD)	0.0000	-	1	
Tissue source site (SD)	0.0333	-	0.02	*
Ship date (SD)	0.0236	-	0.14	
Residual (SD)	0.1477	-	-	

Legend:

- *†: Calculated using the Likelihood Ratio Test between model with versus without variable (e.g. with and without mRNA subtype)*

**: P < 0.05; ***: P < 0.001.*

Supplementary Table S7: Coefficients full linear mixed model (9 tested variables) to explain the variability in estimated tumour purity. Counterpart to the reduced model presented in table 3.

Coefficient	Estimate	Standard error	P value†	
**Fixed effects**			-	
Intercept	75.98	2.20		
VHL mutation/methylation status			0.30	
- VHL methylation	0.58	2.54		
- Moderate impact VHL mutation	-2.52	1.89		
- High impact VHL mutation	1.78	1.60		
- Missing	-0.39	1.45		
Tumour stage			0.06	
- Stage II	1.17	1.77		
- Stage III	-3.18	1.41		
- Stage IV	0.19	1.67		
mRNA subtype			2×10^{-12}	***
- mRNA subtype m2	-3.06	1.68		
- mRNA subtype m3	-12.57	1.69		
- mRNA subtype m4	-4.14	1.72		
- mRNA subtype missing	-0.72	1.64		
Loss of chromosome 3p	-3.87	1.79	0.03	*
Gain of chromosome 5q	-2.76	1.14	0.02	*
Loss of chromosome 14q	-3.42	1.17	0.004	**
Number of chromosomal alterations in p53 and the G1/S cell cycle transition pathway (ranging from 0-15)	0.26	0.16	0.11	
**Random effects**				
Batch ID (SD)	0.79	-	0.70	
Tissue source site (SD)	1.32	-	0.55	
Residual (SD)	11.8	-	-	

Legend:

- *†: Calculated using the Likelihood Ratio Test between model with versus without variable (e.g. with and without mRNA subtype)*
- **: P < 0.05; **: P < 0.01; ***: P < 0.001.*

Supplementary Table S8: Frequency of selected model variables after resampling dataset with replacement for 1,000 times and subsequent backward regression using P < 0.05 as the cutoff for keeping variables in the model.

Model	Variable name	Frequency selected	
Model to explain the variability in mean connectivity score of PX-12 and CAY-10585	Estimated tumour purity	68.9%	∆
	VHL mutation/methylation status	14.9%	
	Tumour stage	32.7%	
	mRNA subtype	95.4%	∆
	Loss of chromosome 3p	100.0%	∆
	Gain of chromosome 5q	96.0%	∆
	Loss of chromosome 14q	70.3%	∆
	Number of chromosomally altered genes which regulate p53 and the G1/S cell cycle transition	100.0%	∆
Model to explain the variability in estimated tumour purity	VHL mutation/methylation status	43.0%	
	Tumour stage	63.2%	∆
	mRNA subtype	99.9%	∆
	Loss of chromosome 3p	61.2%	∆
	Gain of chromosome 5q	69.4%	∆
	Loss of chromosome 14q	83.3%	∆
	Number of chromosomally altered genes which regulate p53 and the G1/S cell cycle transition	27.0%	

Legend:

- *∆: variable selected >50% of the time and therefore kept in final model presented in the main text*
